# Importance of HBsAg recognition by HLA molecules as revealed by responsiveness to different hepatitis B vaccines

**DOI:** 10.1038/s41598-021-82986-8

**Published:** 2021-03-02

**Authors:** Nao Nishida, Masaya Sugiyama, Jun Ohashi, Yosuke Kawai, Seik-Soon Khor, Sohji Nishina, Kazumi Yamasaki, Hirohisa Yazaki, Kaori Okudera, Akihiro Tamori, Yuichiro Eguchi, Aiko Sakai, Keisuke Kakisaka, Hiromi Sawai, Takayo Tsuchiura, Miyuki Ishikawa, Keisuke Hino, Ryo Sumazaki, Yasuhiro Takikawa, Tatsuo Kanda, Osamu Yokosuka, Hiroshi Yatsuhashi, Katsushi Tokunaga, Masashi Mizokami

**Affiliations:** 1 Genome Medical Science Project, National Center for Global Health and Medicine, Ichikawa, 272-8516 Japan; 2 Department of Biological Sciences, Graduate School of Science, The University of Tokyo, Bunkyo-ku, 113-0033 Japan; 3 Department of Hepatology and Pancreatology, Kawasaki Medical School, Okayama, 701-0192 Japan; 4 Clinical Research Center, National Hospital Organization Nagasaki Medical Center, Omura, 856-8562 Japan; 5 Department of Hepatology, Osaka City University Graduate School of Medicine, Osaka, 558-8585 Japan; 6 Division of Hepatology, Saga Medical School, Saga, 840-8502 Japan; 7 Department of Child Health, Faculty of Medicine, University of Tsukuba, Tsukuba, 305-8577 Japan; 8 Division of Hepatology, Department of Internal Medicine, Iwate Medical University, Yahaba-cho, 028-3694 Japan; 9 Department of Human Genetics, Graduate School of Medicine, The University of Tokyo, Bunkyo-ku, 113-0033 Japan; 10 Department of Gastroenterology, Graduate School of Medicine, Chiba University, Chiba, 260-8670 Japan; 11 Division of Gastroenterology and Hepatology, Department of Medicine, Nihon University School of Medicine, Itabashi-ku, 173-8610 Japan; 12 Japan Community Health Care Organization Funabashi Central Hospital, Funabashi, 273-8556 Japan

**Keywords:** Genetics, Biomarkers, Diseases, Gastroenterology, Risk factors

## Abstract

Hepatitis B (HB) vaccines (Heptavax-II and Bimmugen) designed based on HBV genotypes A and C are mainly used for vaccination against HB in Japan. To determine whether there are differences in the genetic background associated with vaccine responsiveness, genome-wide association studies were performed on 555 Heptavax-II and 1193 Bimmugen recipients. Further *HLA* imputation and detailed analysis of the association with *HLA* genes showed that two haplotypes, DRB1*13:02-DQB1*06:04 and DRB1*04:05-DQB1*04:01, were significantly associated in comparison with high-responders (HBsAb > 100 mIU/mL) for the two HB vaccines. In particular, HLA-DRB1*13:02-DQB1*06:04 haplotype is of great interest in the sense that it could only be detected by direct analysis of the high-responders in vaccination with Heptavax-II or Bimmugen. Compared with healthy controls, DRB1*13:02-DQB1*06:04 was significantly less frequent in high-responders when vaccinated with Heptavax-II, indicating that high antibody titers were less likely to be obtained with Heptavax-II. As Bimmugen and Heptavax-II tended to have high and low vaccine responses to DRB1*13:02, 15 residues were found in the Heptavax-II-derived antigenic peptide predicted to have the most unstable HLA-peptide binding. Further functional analysis of selected hepatitis B patients with *HLA* haplotypes identified in this study is expected to lead to an understanding of the mechanisms underlying liver disease.

## Introduction

In Japan, an estimated 1.1–1.4 million individuals (about 1% of the population in Japan) are infected with hepatitis B virus (HBV). Most of these infections were caused by mother-to-child transmission before the start of a nationwide hepatitis B immunization program initiated by the Japanese government in 1986. About 80% of the chronic hepatitis B (CHB) patients in mainland Japan are infected with HBV genotype C, and the remaining are HBV genotype B^1^. The frequency of HBV genotype A in cases of acute hepatitis B (AHB) has been increasing since mid-1990, and HBV genotype A was the predominant genotype for AHB between 2005 and 2010^1^. It has been reported that the number of patients who become chronic after AHB was significantly higher in patients with genotype A than in patients with non-A genotypes^2^. Although a selective HBV vaccination program continued until 2016 in Japan, the Japanese government started routine HBV vaccination for infants from October 2016. Several hepatitis B (HB) vaccines have been developed to prevent HBV infection corresponding to HBV genotypes [i.e. genotype A (Heptavax-II^R^), genotype A2 (Engerix-B^R^; Recombivax HB^R^), or genotype C (Bimmugen^R^)]. In Japan, Bimmugen derived from HBV genotype C has been used in accordance with the background of hepatitis B patients, but in recent years, Heptavax-II derived from HBV genotype A has been increasingly used. Both HB vaccines are given three times, and antibody titers > 100 mIU/mL are considered to provide adequate protection against HBV infection over two years^3,4^. However, the immune response to HB vaccination differs among individuals, with 5–10% of healthy individuals failing to acquire protective levels of antibodies.

There have been several reports in which associations of SNPs in the *HLA* class II region with a response to HB vaccines have been identified, mainly in Asian populations^5–8^. In these previous reports, the studied individuals were vaccinated with HB vaccines designed based on the HBV genotype A2. In our previous report, a genome-wide association study (GWAS) using a total of 1193 Japanese individuals who were vaccinated with Bimmugen revealed the importance of the *HLA-DR-DQ* and *BTNL2* genes for response to an HB vaccine designed based on HBV genotype C^9^. To date, no studies have examined the response of different types of HB vaccines in the same population. Here, we conducted a genomic analysis, i.e. SNP-based GWAS and association tests of *HLA* class II alleles, of Japanese individuals inoculated with Heptavax-II vaccine and investigated the differences between the genetic factors related to Heptavax-II responsiveness and those related to Bimmugen responsiveness.

## Results

### GWAS revealed that *HLA* genes significantly contribute to the responsiveness to both Heptavax-II and Bimmugen

**Figure 1:**
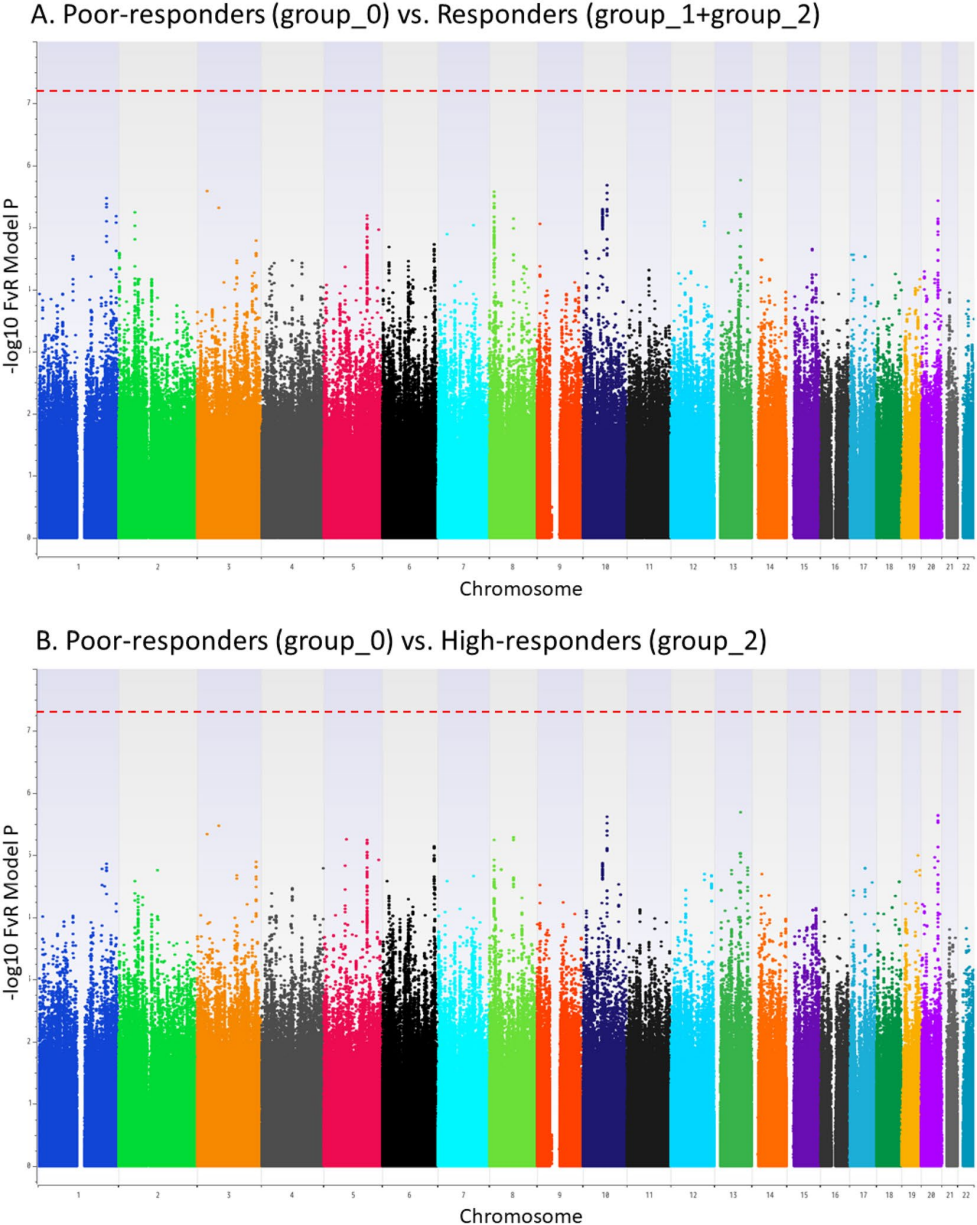
Manhattan plot from dense imputed data by applying a regression analysis with age, sex, and the number of vaccinations as covariates. Among 555 Japanese HB-vaccinated individuals with Heptavax-II, *P* values were calculated in a comparison (**A**) between poor-responders (n = 66) and responders (n = 489), and (**B**) between poor-responders and high-responders (n = 305) using a chi-square test for allele frequencies. Dashed lines indicate genome-wide significance levels of *P* = 5.0 × 10^–8^.

**Figure 2:**
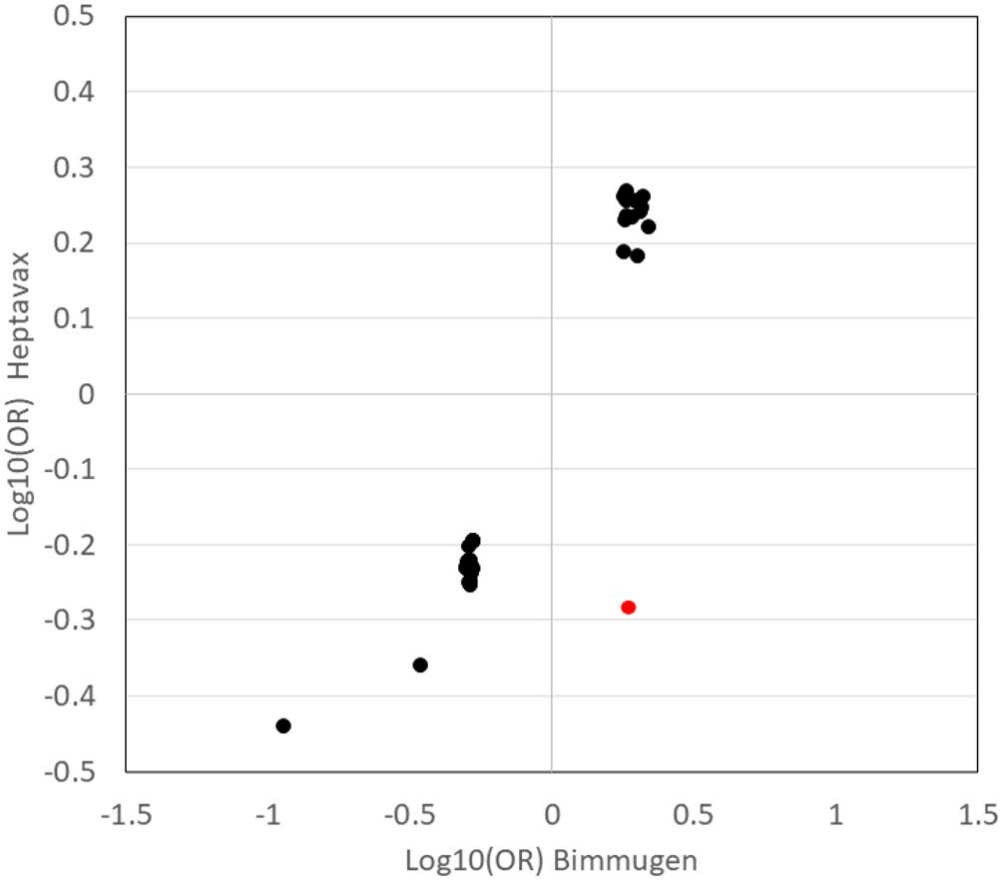
Comparison of odds ratios between Heptavax and Bimmugen for 72 SNPs with *P* < 0.0001 in the Bimmugen GWAS (Group_0 vs. Group_2). Red dot indicates an SNP with opposite OR in the GWAS between Heptavax-II and Bimmugen vaccines (rs12595417, shown in Supplementary Table 3).

Genome-wide multiple regression analysis was carried out to compare poor-responders (i.e. Group_0) and responders (i.e. Group_1 + Group_2) (Supplementary Fig. 1A), and poor responders and high-responders (i.e. Group_2) (Supplementary Fig. 1B), in which age, sex, and the number of vaccinations were used as covariates. No significant association was detected in genome-wide association analysis after genotype imputation (Fig. 1). The SNP rs4248166 present on the *BTNL2* gene, which showed a significant association in the Bimmugen GWAS^9^, was not significantly associated in the Heptavax GWAS (*P* = 0.018). When the OR in a GWAS (Group_0 vs. Group_1 + Group_2) of Heptavax-II was compared to the one in Bimmugen, the top 70 SNPs with P values lower than 0.001 in GWAS of Bimmugen were highly correlated (r^2^ = 0.886) (Fig. 2). Sixty-six SNPs out of 70 were located in the HLA region (Chr.6: 32,570,311–33,080,360, dbSNP b151), and only one SNP, rs12595417, which is located in an intron of long non-coding RNA (LINC02250, Chr.15: 25,793,489), showed an opposite OR in the GWAS between Heptavax-II (OR = 0.52) and Bimmugen vaccines (OR = 1.87) (Supplementary Table 1). A combined association analysis of the cases of Heptavax-II and Bimmugen was subsequently performed, because the genetic background for their responsiveness was found to be similar. Significant associations were detected from the *HLA* region in genome-wide association analysis after genotype imputation in both comparisons between poor-responders and responders (Supplementary Fig. 2A), and between poor-responders and high-responders (Supplementary Fig. 2B). Finally, we carried out genome-wide association analysis after genotype imputation to compare each of the three groups between Heptavax-II and Bimmugen. Although a significant association of rs57190718, located 50 kb downstream from the *PCDH10* gene, was detected when high-responders were compared between Heptavax-II and Bimmugen (Supplementary Fig. 3C), no significant association was found in the validation test by TaqMan assay. The other three SNPs (rs4287780, Chr.2: 166860126 on GRCh38; rs10904760, Chr.10: 16507427 on GRCh38; rs17770803, Chr.15: 24694843 on GRCh38) that met the genome-wide significance level were also genotyped by TaqMan assay, but the associations were not replicated and were therefore revealed to be false positives.

### Two *HLA-DRB1-DQB1* haplotypes showed different responses to Heptavax-II and Bimmugen

Since the GWAS suggested that *HLA* genes play a very important role in responsiveness to either Heptavax-II or Bimmugen vaccines, *HLA* imputation was performed, and *HLA* allele frequencies were compared in detail. We performed statistical imputation of classical *HLA* alleles for three *HLA* loci including *HLA-DRB1*, *DQB1*, and *DPB1* using 555 genome-wide SNP typing data as established in our previous report^10,11^. A total of 515 individuals, all of whom showed high-quality callings in imputation (CT > 0.5) for three *HLA* alleles, were used for the association tests in a comparison between poor-responders and high-responders (Table 1), and between poor-responders and responders (Supplementary Table 2). When poor-responders were compared with high-responders, i.e. excluding intermediate-responders, both HLA-DRB1*04:05-DQB1*04:01 haplotype (OR = 2.09, *P* = 5.84 × 10^–4^) and DPB1*05:01 allele (OR = 2.03, *P* = 3.70 × 10^–4^) were found to have higher ORs than the same haplotype and allele found in a comparison between poor-responders and responders, and these met the significance level. Subsequently, ORs from *HLA* association tests (i.e. poor responders vs. high responders) for Heptavax-II were compared with those for Bimmugen. All 13 *HLA* class II alleles showing *P* < 0.05 in the association tests of Bimmugen showed the same OR trend in both Heptavax-II and Bimmugen, and were highly correlated (r^2^ = 0.842) (Supplementary Table 3). The frequencies of *HLA-DRB1-DQB1* haplotypes and *DPB1* alleles were compared between Heptavax-II and Bimmugen recipients in high-responders (Table 2), responders (Supplementary Table 4), and poor-responders (Supplementary Table 5). The results showed significant associations of two *DRB1-DQB*1 haplotypes, DRB1*04:05-DQB1*04:01 (OR = 1.51, *P* = 2.22 × 10^–3^) and DRB1*13:02-DQB1*06:04 (OR = 0.39, *P* = 3.40 × 10^–4^), in a comparison of high-responders in vaccination with Heptavax-II or Bimmugen. When haplotype frequencies of *HLA-DRB1-DQB1* in high-responders of Heptavax-II and Bimmugen were compared with healthy individuals, the HLA-DRB1*04:05-DQB1*04:01 haplotype (OR = 1.35, *P* = 9.37 × 10^–3^) showed a borderline association, and the HLA-DRB1*13:02-DQB1*06:04 haplotype (OR = 0.42, P = 4.30 × 10^–4^) was significantly less frequent in Heptavax-II recipients (Supplementary Table 6). In contrast, no *DRB1-DQB1* haplotype showed a significant association in Bimmugen recipients.

**Table 1:** Associations of *HLA* class II alleles with a response to the Heptavax-II vaccine in a comparison between poor-responders (Group_0) and high-responders (Group_2).

Haplotype/allele	Group_0		Group_2		Chi P	OR	95% CI	
	Count	%	Count	%			Lower	Upper
DRB1*01:01-DQB1*05:01	3	2.3	41	6.7	4.98E−02	0.32	0.10	1.06
DRB1*04:05-DQB1*04:01	**40**	**30.3**	**105**	**17.2**	**5.84E**−**04**	**2.09**	**1.36**	**3.20**
DRB1*08:03-DQB1*06:01	5	3.8	47	7.7	1.10E−01	0.47	0.18	1.21
DRB1*09:01-DQB1*03:03	16	12.1	98	16.1	2.55E−01	0.72	0.41	1.27
DRB1*13:02-DQB1*06:04	6	4.5	17	2.8	2.91E−01	1.66	0.64	4.30
DRB1*15:01-DQB1*06:02	7	5.3	42	6.9	5.07E−01	0.76	0.33	1.73
DRB1*15:02-DQB1*06:01	14	10.6	78	12.8	4.91E−01	0.81	0.44	1.48
DPB1*02:01	20	15.9	130	23.4	6.62E−02	0.62	0.37	1.04
DPB1*03:01	6	4.8	31	5.6	7.16E−01	0.85	0.35	2.08
DPB1*04:02	6	4.8	54	9.7	7.65E−02	0.46	0.20	1.11
DPB1*05:01	**76**	**60.3**	**238**	**42.8**	**3.70E**−**04**	**2.03**	**1.37**	**3.01**
DPB1*09:01	12	9.5	57	10.3	8.07E−01	0.92	0.48	1.77

The estimated DRB1-DQB1 haplotype frequencies over 5.0% and DPB1 allele frequencies over 5.0% in high-responders (Group_2) are shown. The result of DRB1*13:02-DQB1*06:04 was added for discussion in the paper. The total numbers of poor-responders and high-responders were 63 and 278, respectively, for the DPB1 allele and 66 and 305, respectively, for the DRB1-DQB1 haplotype. Significance level (α) in HLA association analysis was determined by the sorting test, with *P* < 0.00293 for the DRB1-DQB1 haplotype and *P* < 0.00980 for the DPB1 allele. *P* values and odds ratios (OR) were calculated by Pearson’s chi-square test for presence vs. absence of each allele. P values and OR that were statistically significant after correction are indicated in bold.

**Table 2:** Associations of *HLA* class II genes in individuals with a high response (HBsAb > 100 mIU/mL) to Heptavax-II and Bimmugen vaccines.

Haplotype/allele	Heptavax-II		Bimmugen		Chi P	OR	95% CI	
	Count	%	Count	%			Lower	Upper
DRB1*01:01-DQB1*05:01	41	6.7	97	7.1	7.78E−01	0.95	0.65	1.38
DRB1*04:05-DQB1*04:01	**105**	**17.2**	**166**	**12.1**	**2.22E**−**03**	**1.51**	**1.16**	**1.97**
DRB1*08:03-DQB1*06:01	47	7.7	139	10.1	8.73E−02	0.74	0.52	1.05
DRB1*09:01-DQB1*03:03	98	16.1	183	13.3	1.08E−01	1.24	0.95	1.62
DRB1*13:02-DQB1*06:04	**17**	**2.8**	**93**	**6.8**	**3.40E**−**04**	**0.39**	**0.23**	**0.67**
DRB1*15:01-DQB1*06:02	42	6.9	116	8.5	2.34E−01	0.80	0.55	1.16
DRB1*15:02-DQB1*06:01	78	12.8	139	10.1	8.05E−02	1.30	0.97	1.75
DPB1*02:01	130	23.4	342	24.9	4.75E−01	0.92	0.73	1.16
DPB1*03:01	31	5.6	69	5.0	6.24E−01	1.12	0.72	1.72
DPB1*04:02	54	9.7	156	11.4	2.90E−01	0.84	0.60	1.16
DPB1*05:01	238	42.8	525	38.3	6.48E−02	1.21	0.99	1.48
DPB1*09:01	57	10.3	128	9.3	5.33E−01	1.11	0.80	1.54

The estimated DRB1-DQB1 haplotype frequencies over 5.0% and DPB1 allele frequencies over 5.0% in individuals vaccinated with Bimmugen are shown. The total number of high-responders in Heptavax-II and Bimmugen was 278 and 686, respectively, for the DPB1 allele and 305 and 686, respectively, for the DRB1-DQB1 haplotype. Significance level (α) in HLA association analysis was determined by the sorting test, with *P* < 0.00368 for the DRB1-DQB1 haplotype and *P* < 0.00820 for the DPB1 allele. *P* values and odds ratios (OR) were calculated by Pearson’s chi-square test for presence vs. absence of each allele. *P* values and OR that were statistically significant after correction are indicated in bold.

### Prediction of HBsAg peptides recognized by HLA associated with vaccine responsiveness

**Figure 3:**
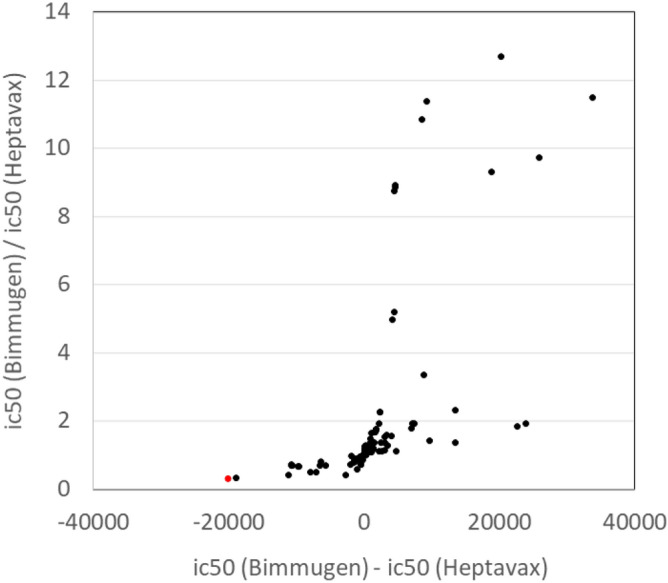
Search for vaccine-derived peptides that are unstable with Heptavax-II and stable with Bimmugen for DRB1*13:02 binding. The horizontal axis plots differences in IC_50_ values of the peptides from Bimmugen and Heptavax-II, and the vertical axis plots the ratio of IC_50_. The red dot indicates the most promising candidate for instability among Heptavax-II-derived peptides and stability among Bimmugen-derived peptides.

Two *HLA-DRB1-DQB1* haplotypes (DRB1*04:05-DQB1*04:01 and DRB1*13:02-DQB1*06:04) that were significantly associated with high vaccine response in a comparison of Heptavax-II and Bimmugen recipients were predicted to bind vaccine-derived antigens. Peptide predictions were performed for 226 aa of HB vaccines (both Heptavax-II and Bimmugen) that bind to DRB1*04:05 and DRB1*13:02 using IEDB epitope analysis (Supplementary Table 7). Binding coefficients were estimated for a total of 212 peptides for both Heptavax-II and Bimmugen, 120 of which had different binding coefficients (IC_50_) between Heptavax-II and Bimmugen. The predicted bindings to DRB1*04:05 and DRB1*13:02 were performed using the NetMHCII 1.1 method, and the top 10 peptides with small IC_50_ (that is, HLA-peptide binding is strong) are summarized in Supplementary Tables 8 and 9, respectively. The frequency of the DRB1*13:02 allele was significantly lower in the group with a high response to Heptavax-II inoculation, suggesting that Heptavax-II-derived HBsAg peptide has unstable binding to DRB1*13:02. To search for vaccine-derived peptides that are unstable in Heptavax-II and stable in Bimmugen to bind DRB1*13:02, the IC_50_ values of peptides having the same position were compared (Fig. 3). One peptide consisting of 15 amino acids at positions 134–148 differed in the 10th amino acid between Heptavax-II and Bimmugen, and the binding of the Bimmugen-derived peptide, which has serine (S) at position 144 (FPSCCCTKP**S**DGNCT), was most significantly destabilized by substitution with threonine (T) in Heptavax-II (FPSCCCTKP**T**DGNCT) to HLA-DRB1*13:02.

## Discussion

In SNP-based association analysis, no SNP significantly associated with a response to the Heptavax-II vaccine. However, the genetic factors involved in the responsiveness to both Heptavax-II and Bimmugen vaccines were found to be very similar. Seventy SNPs with P values less than 0.0001 in the GWAS of 1,193 Bimmugen recipients were highly correlated, with r^2^ = 0.8856, when compared with the odds ratios obtained in the GWAS of 555 Heptavax-II recipients. Of the 70 SNPs, 66 were located in the *HLA* class II region, and only 1 (rs12595417 located on the LINC02250 gene) had the OR reversed. Although rs12595417 did not reach a genome-wide significance level in either Heptavax-II or Bimmugen GWAS (*P* = 1.82 × 10^−5^ and *P* = 1.87 × 10^−3^, respectively), it is present in a multigenic region containing the *SNHG14* gene, which has been reported to be associated with the progression of cervical^12^ and lung cancer^13^, *UBE3A*, which is known to be critical for the development of human papillomavirus-associated cancers^14^, and *ATP10A* (also known as *ATP10C*), which has been reported to encode a putative aminophospholipid translocase associated with Angelman syndrome^15^. Because the ORs of SNPs with low P values in the GWAS of Heptavax-II and Bimmugen were highly correlated, the combined GWAS including both Heptavax-II and Bimmugen and comparisons of each of the three groups (poor-responders, responders, and high-responders) between Heptavax-II and Bimmugen was performed. The combined GWAS showed that *HLA* class II genes contribute significantly to hepatitis B vaccine responsiveness.

In order to understand the contribution of *HLA* class II genes to vaccine responsiveness in more detail, we carried out *HLA* imputation to determine *HLA* alleles, and performed *HLA* association tests. As a result of *HLA* association tests, HLA-DRB1*04:05-DQB1*04:01 haplotype and DPB1*05:01 allele showed significant association with Heptavax-II responsiveness. These *HLA* class II genes were the same as those that we previously reported showing a significant association in Bimmugen responsiveness^9^. Therefore, we compared the ORs of *HLA* class II alleles that were significant in *HLA* association analysis of Bimmugen, and found a very high correlation between Heptavax-II and Bimmugen (r^2^ = 0.8419). In addition, *HLA* allele/haplotype frequencies were compared in each of the three groups. Unexpectedly, two *DRB1-DQB1* haplotypes, DRB1*04:05-DQB1*04:01 and DRB1*13:02-DQB1*06:04, were significantly associated in a comparison of high-responders (*P* = 2.22 × 10^–3^, *P* = 3.40 × 10^–4^, respectively).

HLA-DRB1*04:05-DQB1*04:01 was significantly associated with vaccine hypo-responsiveness in both Heptavax-II and Bimmugen recipients (OR = 2.09 and OR = 2.85, respectively). From the greater OR for Bimmugen, it can be understood that individuals with HLA-DRB1*04:05-DQB1*04:01 are more likely to have a significantly lower vaccine response with Bimmugen than with Heptavax-II. As a result of the binding prediction of HBsAg peptide to DRB1*04:05 by IEDB, there were many peptides predicted only for Heptavax-II that bind more stably than those predicted only for Bimmugen (i.e. low IC_50_). It can be understood that due to the presence of many HBsAg peptides derived from Heptavax-II that bind stably to DRB1*04:05, DRB1*04:05 is an allele that contributes to vaccine hypo-responsiveness but to a lesser extent than to Bimmugen.

HLA-DRB1*13:02-DQB1*06:04 was a haplotype that was not significantly associated with vaccine responsiveness in either Heptavax-II or Bimmugen recipients (*P* = 2.91 × 10^–1^, *P* = 1.09 × 10^–1^, respectively). However, when high-responders in both vaccinations were compared, the frequency of the HLA-DRB1*13:02-DQB1*06:04 haplotype was significantly lower in the Heptavax-II group. Although the difference was not significant, focusing on the ORs in Heptavax-II-vaccinated and Bimmugen-vaccinated individuals, the haplotype was associated with a tendency for low vaccine responses in Heptavax-II (OR = 1.66) and high vaccine responses in Bimmugen (OR = 0.53). From this result, it can be understood that individuals with the HLA-DRB1*13:02-DQB1*06:04 haplotype can obtain higher antibody titers (HBsAb > 100 mIU/mL) by being vaccinated with Bimmugen rather than with Heptavax-II. In the binding prediction of vaccine-derived antigen peptides to the HLA-DRB1*13:02 allele by IEDB, a peptide that is unstable for Heptavax-II but is stable for Bimmugen was found. The 15 residues from position 134–148 (FPSCCCTKP [T/S] DGNCT) are the most suitable for binding.

A previous report in Japan has examined whether Bimmugen recipients are protected against HBV genotype A infection and whether Heptavax-II recipients are protected against HBV genotype C infection^16^. The results showed that sufficient antibody titers (i.e. HBsAb > 100 mIU/mL) could protect against infection from different HBV genotypes. Our findings showed that HBsAb > 100 mIU/mL is less likely to be obtained in individuals with the HLA-DRB1*13:02-DQB1*06:04 haplotype when vaccinated with Heptavax-II, indicating that Bimmugen vaccination is preferred for these individuals.

As we reported previously, HLA-DRB1*13:02-DQB1*06:04 is a significantly less frequent haplotype in patients with chronic hepatitis B^17^ (Table 3). Taken together with the reverse contribution of Heptavax-II and Bimmugen to vaccine responsiveness, it can be understood that HBsAg peptides common to Heptavax-II and Bimmugen do not contribute to disease susceptibility. Approximately 80% of hepatitis patients in Japan are infected with HBV genotype C, but the mechanism by which HLA molecules contribute to disease resistance remains unclear. If HLA-DRB1*13:02-DQB1*06:04 is significantly associated with a high vaccine response in Bimmugen recipients from the same HBV genotype C, it may be demonstrated that recognition of Bimmugen-derived HBsAg peptide by HLA-DRB1*13:02-DQB1*06:04 plays an important role in disease resistance.

**Table 3:** Haplotype frequencies and genotype counts in Japanese vaccines (high-responders), Japanese chronic hepatitis B patients, and healthy Japanese individuals.

	DRB1*13:02-DQB1*06:04	
	Frequencies	Genotype counts
Heptavax-II recipients	2.8%*	17/610
Bimmugen recipients	6.8%	93/1372
HBV patients	2.3%**	37/1630
Healthy individuals	6.4%	291/4562

*Significant association was identified in comparison with healthy Japanese individuals (OR = 0.34, *P* = 4.30 × 10^−4^).

**Significant association was identified in comparison with healthy Japanese individuals (OR = 0.42, *P* = 2.05 × 10^−10^).

In conclusion, genomic analyses of a Japanese population vaccinated with different hepatitis vaccines (Heptavax-II and Bimmugen) revealed that there are specific *HLA-DRB1-DQB1* haplotypes with different responses, although the genetic factors involved in vaccine responsiveness are very similar. Further functional analysis of selected hepatitis B patients with *HLA* haplotypes identified in this study is expected to lead to an understanding of the mechanisms underlying liver disease.

## Materials and methods

### Ethics approval

This study was approved by the Ethics Committee of the National Center for Global Health and Medicine and of all of the following Institutes and Hospitals throughout Japan that participated in this collaborative study: the University of Tokyo; Kawasaki Medical School; National Hospital Organization Nagasaki Medical Center; Osaka City University; Saga University; the University of Tsukuba; Iwate Medical University; and Chiba University. All participants provided written informed consent for participation in this study, and the methods were carried out in accordance with the approved guidelines.

### Samples and clinical data

New cases vaccinated with a recombinant absorbed HB vaccine (Heptavax-II^R^, MSD KK, Tokyo, Japan) were collected under conditions similar to those used in our previous study using individuals vaccinated with the Bimmugen^R^ vaccine (Kaketsuken, Kumamoto, Japan)^9^. All 555 Japanese genomic DNA samples were obtained from healthy adult volunteers (≥ 18 years) who were vaccinated in three doses (0.5 mL) at 0, 1, and 6 months with the Heptavax-II vaccine. Only those who received all three doses of the Heptavax-II vaccine were included in this study. Serum anti-HBV surface antibody (HBsAb) and serum anti-HBV core antibody (HBcAb) were tested before vaccination and at 1 month after final inoculation using the ARCHITECT anti-HBs kit (Abbott Japan, Tokyo, Japan) and the ARCHITECT anti-HBc II kit (Abbott Japan, Tokyo, Japan), respectively, with a fully automated chemiluminescent enzyme immunoassay system using the Architect i2000SR analyzer (Abbott Japan, Tokyo, Japan). Individuals who were HBcAb-positive (> 1.0 S/CO) were not included in this study. Although there has been a report of an HBV vaccinated donor showing an HBsAb value of 96 IU/L who was found to be positive for HBV DNA, we set levels of antibodies against HBV surface antigen as 10 IU/L or more to be considered as having immunity against HBV, following the World Health Organization recommendation^4^. In this study, we categorized 555 individuals into three groups: Group_0, poor-responders, HBsAb ≤ 10 mIU/mL; Group_1, intermediate-responders, 10 mIU/mL < HBsAb < 100 mIU/mL; Group_2, high-responders, HBsAb ≥ 100 mIU/mL. The clinical information of 555 individuals is summarized for each group in Supplementary Table 10. Data of age and number of past vaccinations with Heptavax-II were collected from all 555 individuals using a questionnaire. Data of computed sex for the 555 individuals were acquired from the genome-wide SNP genotyping data of the AXIOM genome-Wide ASI 1 array (Thermo Fisher Scientific, Inc., Waltham, MA, USA) acquired in this study. The significant associations of sex and age with HBsAb levels were identified by simple linear regression analysis (*P* = 2.32 × 10^−3^ and *P* = 2.30 × 10^−3^, respectively). In contrast, the number of past vaccinations with Heptavax-II showed no association in simple linear regression analysis (*P* = 0.82).

A modified table including clinical information about 1193 individuals previously reported to be vaccinated with Bimmugen is summarized in Supplementary Table 11.

### Genome-wide SNP genotyping in individuals vaccinated with Heptavax-II

For the GWAS, the 555 Japanese genomic DNA samples from individuals vaccinated with Heptavax-II were genotyped using the Axiom Genome-Wide ASI 1 array, according to the manufacturer's instructions. The following analysis was performed according to the same way as the analysis in our previous GWAS on Bimmugen^9^. All samples had an overall call rate of more than 95%; the average overall call rate was 99.41% (95.01–99.93) and passed a heterozygosity check. No related individual (PI ≥ 0.1) was identified in identity-by-descent testing. Principal component analysis was carried out to check the genetic background in the studied 555 samples together with HapMap samples (43 JPT, 40 CHB, 91 YRI, and 91 CEU samples). This analysis showed that all 555 samples formed the same cluster using the first and second components, indicating that the effect of population stratification was negligible in the studied samples (Supplementary Fig. 4).

### SNP genotype imputation and association test

For high-density mapping following the GWAS, we carried out SNP genotype imputation from 555 Heptavax-II and 1193 Bimmugen recipients by Beagle version 4.1 (URL: https://faculty.washington.edu/browning/beagle/b4_1.html) using the 1000 genomes as the imputation reference^18^. The following thresholds were then applied for SNP quality control during the data cleaning: SNP call rate ≥ 95%; minor allele frequency ≥ 5%; and Hardy–Weinberg Equilibrium *P* value ≥ 0.001. Of the SNPs on autosomal chromosomes, 5,835,420 SNPs finally passed the quality control filters and were used for genome-wide multiple regression analysis using age, sex, and the number of vaccinations as covariates. Figure 1 shows association analysis using 555 Japanese HB vaccinated individuals with Heptavax-II in a comparison between poor-responders (Group_0) and responders (Group_1 + Group_2) (Fig. 1A), and between poor-responders and high-responders (Group_2) (Fig. 1B). A combined analysis of Heptavax-II (n = 555) and Bimmugen (n = 1193) (Supplementary Fig. 2), and a comparison of each group (i.e. Group_0, Group_1, and Group_2) to two different vaccines (Supplementary Fig. 3), were carried out. To avoid false positives due to multiple testing, genome-wide significance was set at *P* < 5.0 × 10^−8^. Four SNPs satisfying the significance level in a comparison of Group_2 between Heptavax-II and Bimmugen were validated to see if the association could be reproduced by the TaqMan assay (Thermo Fisher Scientific Inc., MA, USA).

### HLA imputation

SNP data from 555 samples were extracted from an extended MHC (xMHC) region ranging from 25,759,242 to 33,534,827 bp based on the hg19 position. We conducted 2-field *HLA* genotype imputation for three class II *HLA* genes using the HIBAG R package as the same way in our previous report^9^. For *HLA-DRB1*, *DQB1*, and *DPB1*, our in-house Japanese imputation reference was used for *HLA* genotype imputation^10,11^. We applied post-imputation quality control using call-threshold (CT > 0.5). A total of 515 samples consisting of 63 in Group_0, 174 in Group_1, and 278 in Group_2 showed three estimated *HLA* genotypes, all of which met the threshold. In total, we imputed 22 *HLA-DRB1*, 14 *HLA-DQB1*, and 11 *HLA-DPB1* genotypes for *HLA* class II genes.

### Haplotype estimation

Haplotype phasing was performed in a manner similar to that performed in the previous analysis of Bimmugen^9^. The phased haplotypes consisting of three *HLA* class II loci (*HLA-DRB1*, *DQB1*, and *DPB1*) were estimated using the PHASE program version 2.1 (URL: http://stephenslab.uchicago.edu/index.html)^19^. The estimated 3-locus haplotypes were further used for the estimation of haplotypes of *HLA-DRB1* and *DQB1* loci (i.e., the collapsing method was applied to the phased data for three *HLA* loci). See our previous paper for more details^9^.

### *HLA* association test

To assess the association of *HLA* allele or haplotype with a response to HB vaccination, P values and odds ratios (OR) were calculated by Pearson’s chi-square test in the presence vs. absence of each allele or haplotype. In Table 1 and Supplementary Table 2, *HLA* association analysis using 555 Japanese HB vaccinated individuals with Heptavax-II was carried out in a comparison between poor-responders (Group_0) and high-responders (Group_2), and between poor-responders (Group_0) and responders (Group_1 + Group_2). *HLA* association analysis between Heptavax-II and Bimmugen recipients was carried out in high-responders, responders, and poor-responders (Table 2, Supplementary Tables 4 and 5, respectively). *HLA* frequencies of *DRB1-DQB1* haplotypes were compared between healthy individuals and high-responders in Heptavax-II and Bimmugen inoculation (Supplementary Table 6). The healthy individuals used here were the same as those used in our previous study^17^.

To avoid false positives due to multiple tests, the family-wise error rate was set to be less than 0.05 in the association analysis. The threshold of the *P* value for each allele frequency table or haplotype frequency table was determined by the permutation test.

### Prediction of HBV-related peptide

Peptide predictions were performed for 226 amino acids (aa) of HB vaccines (Heptavax-II and Bimmugen) using the immune epitope database and analysis resource (IEDB: https://www.iedb.org/). Combinatorial library (CombLib), NetMHCII 1.1 (SMM_align), NetMHCII 2.3 (NN_align), NetMHCIIpan 3.2, and Sturniolo methods were used to perform prediction analyses for MHC-II binding. Epitopes that were assigned an IC_50_ value lower than 500 and/or percentile rank lower than 2 were considered as high affinity binders. Both Heptavax-II and Bimmugen predicted that all top 10 peptides bound strongly to DRB1*04:05, whereas they all bound weakly to DRB1*13:02, with IC_50_ ≥ 500 and rank ≥ 2.

### Web resources

GWAS data in this study will be submitted to the NBDC Human Database, a public database in Japan (NBDC Human Database, https://humandbs.biosciencedbc.jp/en/).

## Supplementary Information

Supplementary Information.
