# Supplementary material for: Importance of HBsAg recognition by HLA molecules as revealed by responsiveness to different hepatitis B vaccines
Source: Sci Rep. 2021 Mar 2;11:3703. doi: 10.1038/s41598-021-82986-8 (PMC7925550; doi:10.1038/s41598-021-82986-8)
Supplement: Supplementary file 1 — Supplementary Information. [file 41598_2021_82986_MOESM1_ESM.docx]

**SUPPLEMENTARY MATERIAL**

Importance of HBsAg recognition by HLA molecules as revealed by responsiveness to different hepatitis B vaccines

Nao Nishida, Masaya Sugiyama, Yosuke Kawai, Seik-Soon Khor, Jun Ohashi, Sohji Nishina, Kazumi Yamasaki, Hirohisa Yazaki, Kaori Okudera, Akihiro Tamori, Yuichiro Eguchi, Aiko Sakai, Keisuke Kakisaka, Hiromi Sawai, Takayo Tsuchiura, Miyuki Ishikawa, Keisuke Hino, Ryo Sumazaki, Yasuhiro Takikawa, Tatsuo Kanda, Osamu Yokosuka, Hiroshi Yatsuhashi, Katsushi Tokunaga, and Masashi Mizokami

Supplementary Figure 1. Genome-wide association results applying a regression analysis with age, sex, and the number of vaccinations as covariates. Among 555 Japanese HB-vaccinated individuals with Heptavax-II, P-values were calculated in a comparison (A) between poor-responders (n=66) and responders (n=489), and (B) between poor-responders and high-responders (n=305) using a chi-square test for allele frequencies. Dashed lines indicate genome-wide significance levels of P=5.0×10^-8^.


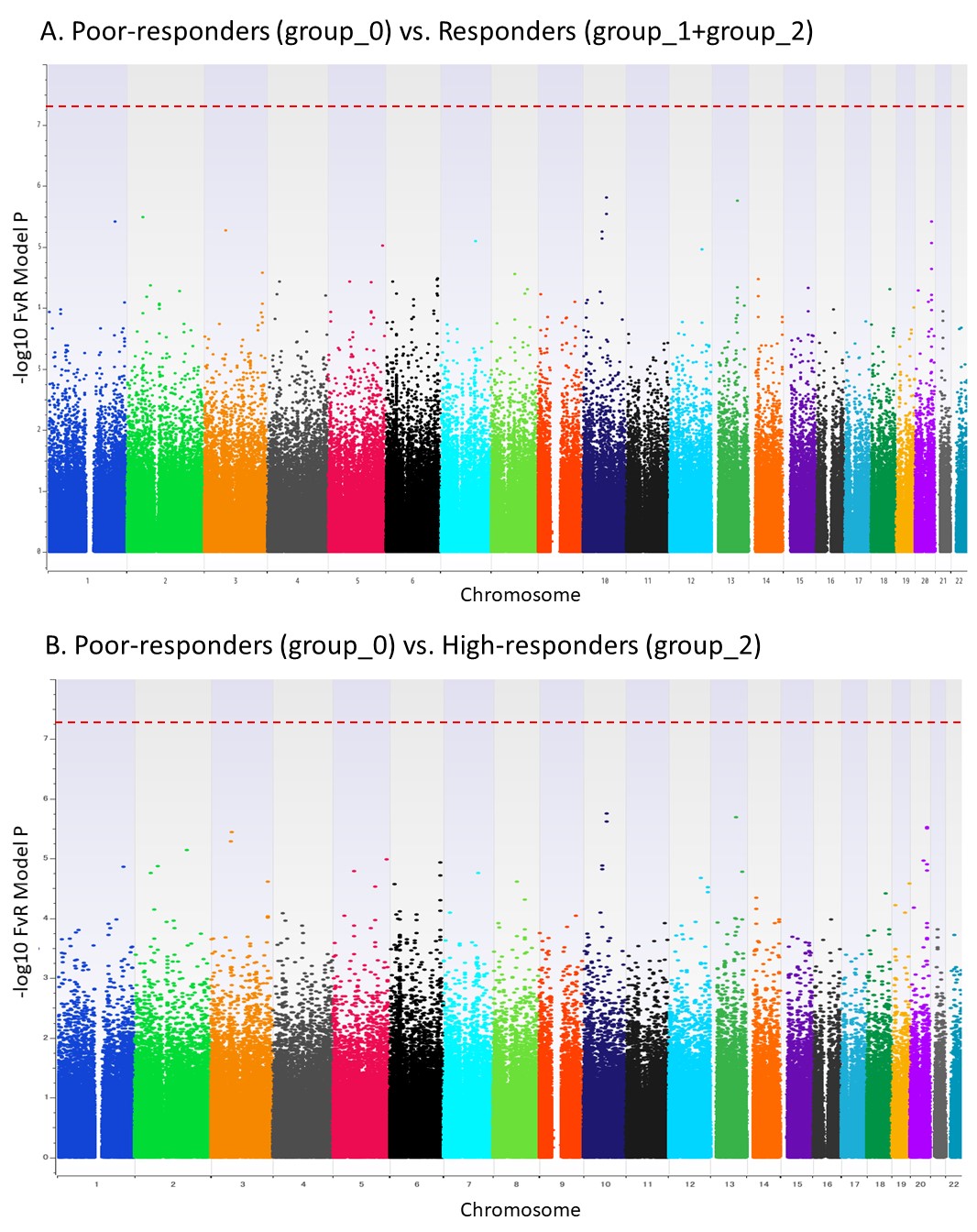


Supplementary Figure 2. Manhattan plot from dense imputed data by applying a regression analysis with age, sex, and the number of vaccinations as covariates. P-values were calculated for the combined cases of Heptavax-II (n=555) and Bimmugen (n=1,193) in a comparison (A) between poor-responders (n=66+107) and responders (n=489+1986), and (B) between poor-responders and high-responders (n=305+735) using a chi-square test for allele frequencies. Dashed lines indicate genome-wide significance levels of P=5.0×10^-8^.


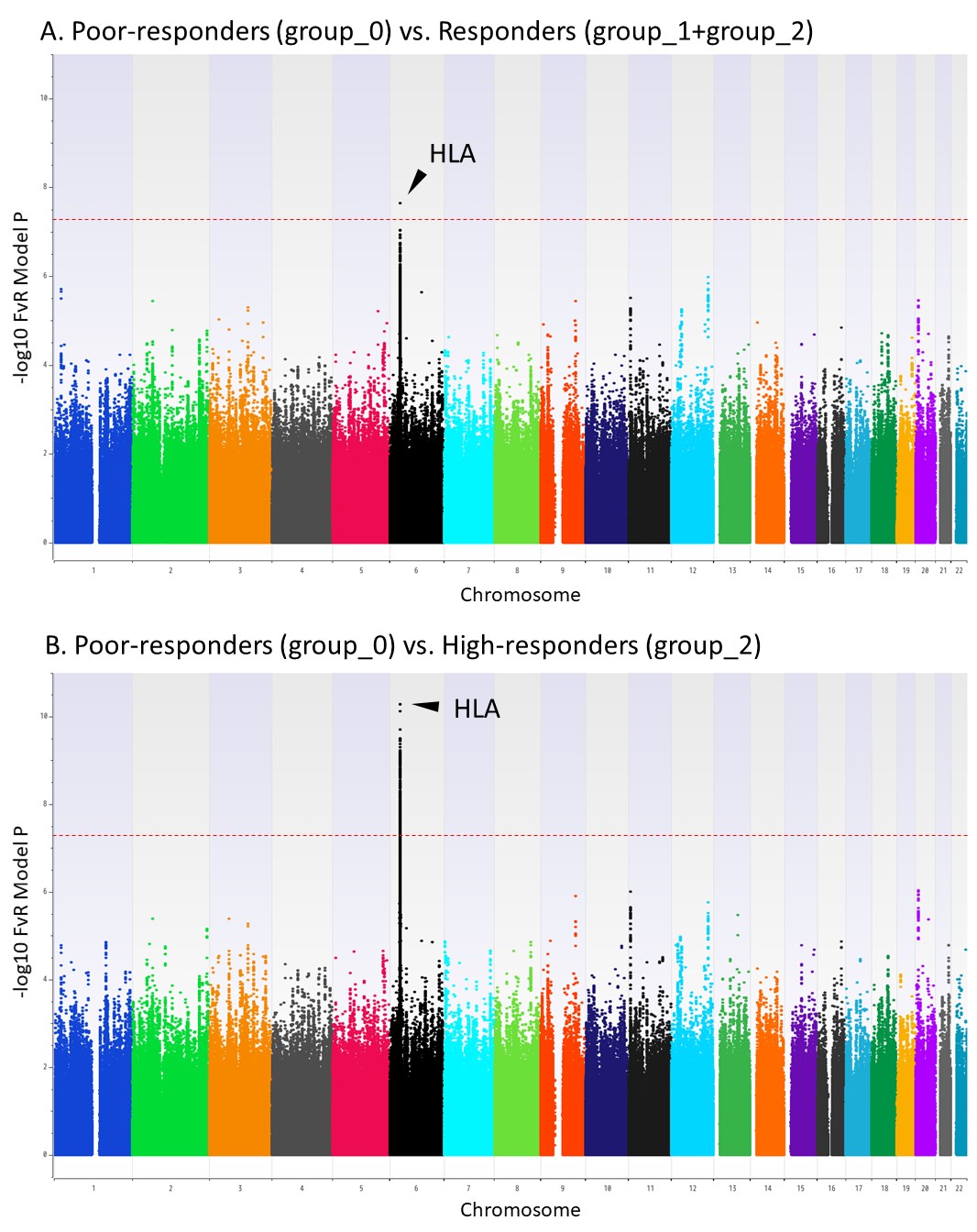


Supplementary Figure 3. Manhattan plot from dense imputed data by applying a regression analysis with age, sex, and the number of vaccinations as covariates. P-values were calculated in a comparison between individuals vaccinated with Heptavax-II (n=555) and ones with Bimmugen (n=1,193) for (A) poor-responders (group_0), (B) intermediate responders (group_1), and (C) high-responders (group_2), using a chi-square test for allele frequencies. Dashed lines indicate genome-wide significance levels of P=5.0×10^-8^.


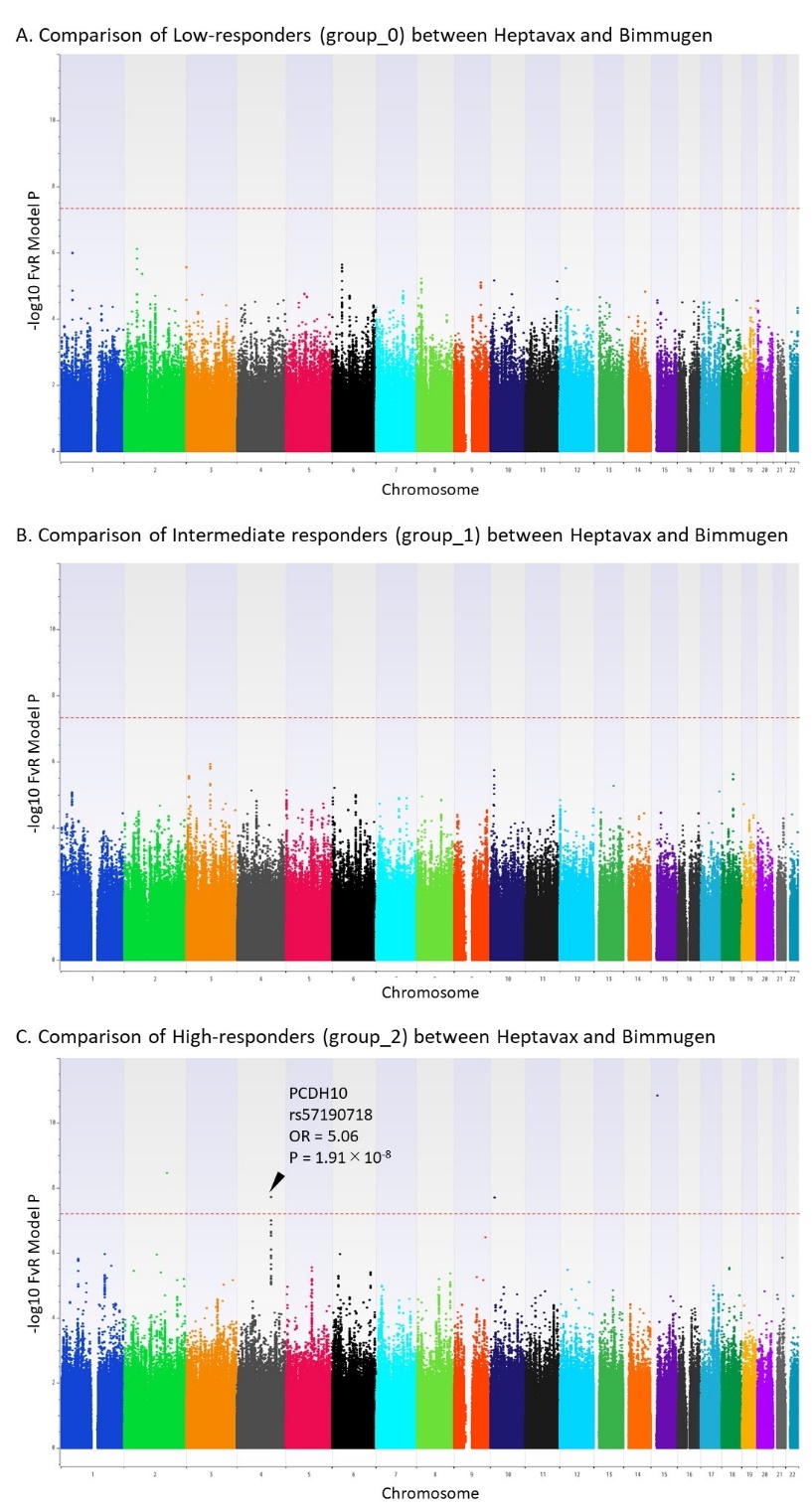


Supplementary Figure 4. Principal component analysis of 555 samples used in the GWAS together with HapMap samples (43 JPT, 40 CHB, 91 YRI, and 91 CEU samples). (A) all groups, (B) focused on Asian samples (studied samples, JPT, and CHB samples).


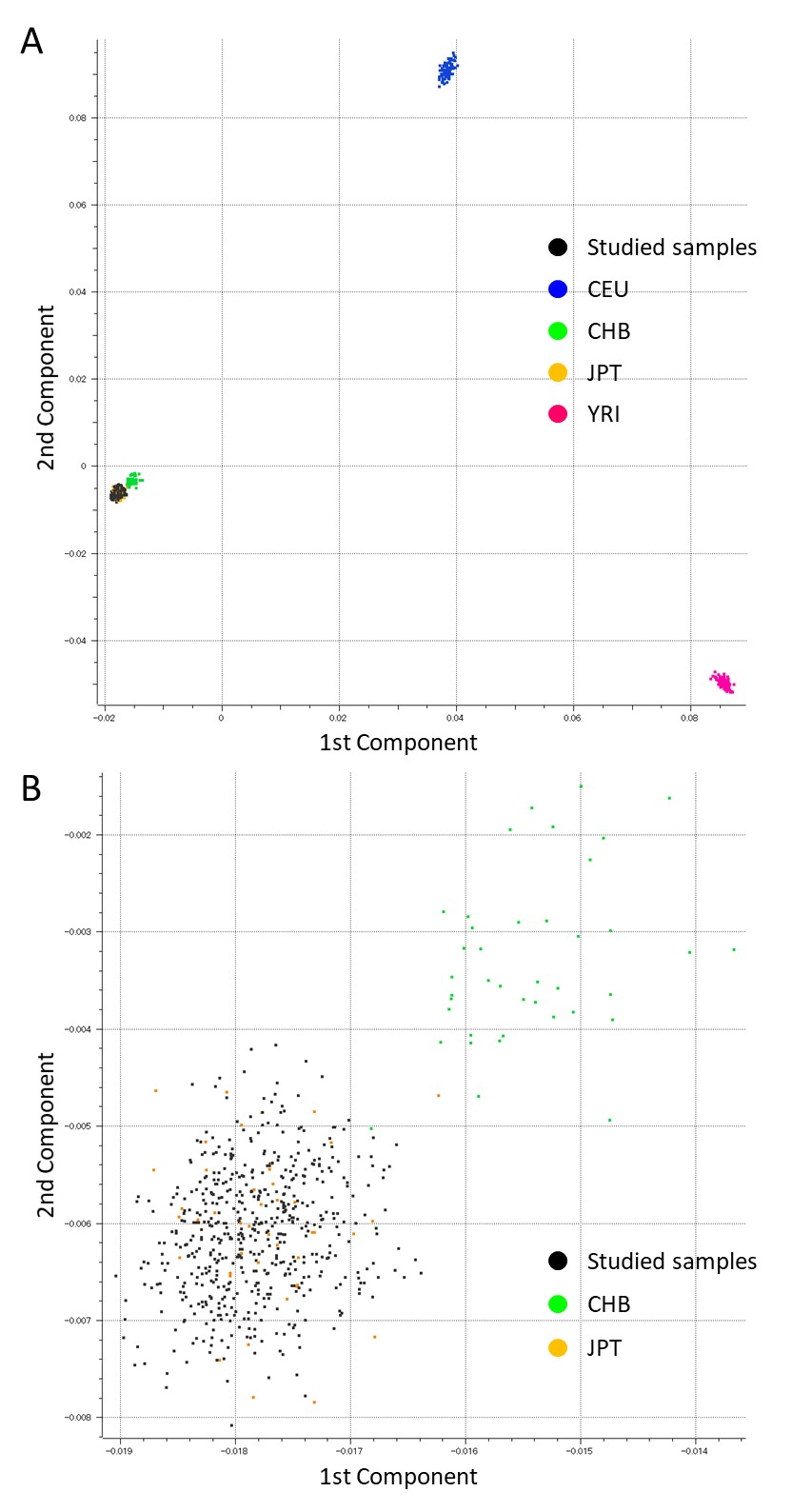


Supplementary Table 1. Comparison of odds ratios between Heptavax and Bimmugen GWAS for 70 SNPs with P <0.0001 in the Bimmugen GWAS (Group_0 vs. Group_1+Group_2)

| rsID | Chr | Position (dbSNP b151) | Associated Gene | Bimmugen GWAS | | Heptavax-II GWAS | |
| --- | --- | --- | --- | --- | --- | --- | --- |
|  |  |  |  | P-Value | Odds Ratio | P-Value | Odds Ratio |
| rs34039593 | 6 | 32570311 | HLA-DQA1 | 4.22E-07 | 2.18 | 1.41E-02 | 1.67 |
| rs614348 | 6 | 32573877 | HLA-DQA1 | 1.13E-06 | 2.10 | 2.91E-03 | 1.82 |
| rs482205 | 6 | 32576009 | HLA-DQA1 | 2.91E-06 | 2.05 | 6.39E-03 | 1.74 |
| rs481139 | 6 | 32576145 | HLA-DQA1 | 2.14E-06 | 2.07 | 5.32E-03 | 1.77 |
| rs660895 | 6 | 32577380 | HLA-DQA1 | 9.59E-05 | 1.79 | 2.80E-02 | 1.54 |
| rs7770370 | 6 | 33048921 | HLA-DPB1 | 9.20E-06 | 1.96 | 3.05E-03 | 1.80 |
| rs9378176 | 6 | 33049309 | HLA-DPB1 | 2.71E-05 | 1.87 | 6.23E-03 | 1.71 |
| rs9378177 | 6 | 33049384 | HLA-DPB1 | 1.67E-05 | 1.91 | 5.99E-03 | 1.72 |
| rs9277357 | 6 | 33049979 | HLA-DPB1 | 8.70E-05 | 0.53 | 3.06E-02 | 0.64 |
| rs9277361 | 6 | 33050045 | HLA-DPB1 | 9.36E-05 | 0.53 | 3.06E-02 | 0.64 |
| rs9277362 | 6 | 33050078 | HLA-DPB1 | 9.06E-05 | 0.53 | 3.06E-02 | 0.64 |
| rs9277378 | 6 | 33050279 | HLA-DPB1 | 9.18E-05 | 0.53 | 3.06E-02 | 0.64 |
| rs9277382 | 6 | 33050441 | HLA-DPB1 | 9.34E-05 | 0.53 | 2.98E-02 | 0.64 |
| rs3128960 | 6 | 33050654 | HLA-DPB1 | 8.17E-05 | 0.53 | 3.06E-02 | 0.64 |
| rs9277393 | 6 | 33050877 | HLA-DPB1 | 6.26E-05 | 0.51 | 1.41E-02 | 0.60 |
| rs9277395 | 6 | 33051051 | HLA-DPB1 | 5.29E-05 | 0.51 | 1.36E-02 | 0.59 |
| rs9277396 | 6 | 33051139 | HLA-DPB1 | 7.15E-05 | 0.52 | 1.17E-02 | 0.59 |
| rs9277410 | 6 | 33051640 | HLA-DPB1 | 5.70E-05 | 0.51 | 7.16E-03 | 0.56 |
| rs9277411 | 6 | 33051683 | HLA-DPB1 | 5.39E-05 | 0.51 | 1.20E-02 | 0.59 |
| rs9277412 | 6 | 33051689 | HLA-DPB1 | 6.70E-05 | 0.52 | 1.60E-02 | 0.60 |
| rs9277413 | 6 | 33051720 | HLA-DPB1 | 5.48E-05 | 0.51 | 1.22E-02 | 0.59 |
| rs9277418 | 6 | 33051749 | HLA-DPB1 | 5.24E-05 | 0.51 | 1.23E-02 | 0.59 |
| rs9277421 | 6 | 33051820 | HLA-DPB1 | 7.42E-05 | 0.52 | 9.55E-03 | 0.58 |
| rs9277424 | 6 | 33051865 | HLA-DPB1 | 5.79E-05 | 0.51 | 1.30E-02 | 0.59 |
| rs9277429 | 6 | 33052007 | HLA-DPB1 | 4.94E-05 | 0.51 | 6.64E-03 | 0.56 |
| rs9277434 | 6 | 33052186 | HLA-DPB1 | 5.00E-05 | 0.51 | 1.31E-02 | 0.59 |
| rs9277437 | 6 | 33052250 | HLA-DPB1 | 5.17E-05 | 0.51 | 1.22E-02 | 0.59 |
| rs9277441 | 6 | 33052354 | HLA-DPB1 | 4.55E-05 | 0.51 | 2.78E-02 | 0.63 |
| rs9277458 | 6 | 33053167 | HLA-DPB1 | 6.65E-05 | 0.52 | 1.14E-02 | 0.59 |
| rs9277463 | 6 | 33053307 | HLA-DPB1 | 9.23E-05 | 0.53 | 1.12E-02 | 0.59 |
| rs9277464 | 6 | 33053352 | HLA-DPB1 | 4.14E-05 | 0.51 | 1.65E-02 | 0.60 |
| rs9277466 | 6 | 33053399 | HLA-DPB1 | 4.71E-05 | 0.51 | 1.41E-02 | 0.60 |
| rs9277471 | 6 | 33053682 | HLA-DPB1 | 4.61E-05 | 0.51 | 1.22E-02 | 0.59 |
| rs9277489 | 6 | 33053942 | HLA-DPB1 | 6.54E-05 | 0.51 | 1.36E-02 | 0.59 |
| rs9277508 | 6 | 33054177 | HLA-DPB1 | 6.79E-05 | 0.52 | 1.31E-02 | 0.59 |
| rs9277509 | 6 | 33054207 | HLA-DPB1 | 4.96E-05 | 0.51 | 1.22E-02 | 0.59 |
| rs9277517 | 6 | 33054281 | HLA-DPB1 | 4.45E-05 | 0.51 | 1.12E-02 | 0.59 |
| rs1042544 | 6 | 33054457 | HLA-DPB1 | 4.79E-05 | 0.51 | 1.23E-02 | 0.59 |
| rs9277533 | 6 | 33054721 | HLA-DPB1 | 7.88E-05 | 0.52 | 1.10E-02 | 0.58 |
| rs9277534 | 6 | 33054807 | HLA-DPB1 | 5.20E-05 | 0.51 | 1.22E-02 | 0.59 |
| rs9277535 | 6 | 33054861 | HLA-DPB1 | 3.31E-05 | 0.50 | 1.22E-02 | 0.59 |
| rs9277536 | 6 | 33054890 | HLA-DPB1 | 4.51E-05 | 0.51 | 1.14E-02 | 0.59 |
| rs9277537 | 6 | 33055009 | HLA-DPB2 | 3.94E-05 | 0.51 | 1.06E-02 | 0.58 |
| rs9277542 | 6 | 33055247 | HLA-DPB2 | 5.30E-05 | 0.51 | 1.40E-02 | 0.59 |
| rs9277546 | 6 | 33055346 | HLA-DPB2 | 5.23E-05 | 0.51 | 1.22E-02 | 0.59 |
| rs9277549 | 6 | 33055419 | HLA-DPB2 | 3.30E-05 | 0.50 | 1.36E-02 | 0.59 |
| rs3128963 | 6 | 33055780 | HLA-DPB2 | 4.91E-05 | 0.51 | 1.22E-02 | 0.59 |
| rs3128964 | 6 | 33055818 | HLA-DPB2 | 4.34E-05 | 0.51 | 1.14E-02 | 0.59 |
| rs3117229 | 6 | 33056069 | HLA-DPB2 | 3.44E-05 | 0.50 | 1.22E-02 | 0.59 |
| rs3130186 | 6 | 33056207 | HLA-DPB2 | 3.14E-05 | 0.50 | 1.22E-02 | 0.59 |
| rs3130187 | 6 | 33056405 | HLA-DPB2 | 2.85E-05 | 0.50 | 1.22E-02 | 0.59 |
| rs3117228 | 6 | 33056435 | HLA-DPB2 | 2.86E-05 | 0.50 | 1.14E-02 | 0.59 |
| rs3091281 | 6 | 33056566 | HLA-DPB2 | 3.38E-05 | 0.50 | 1.30E-02 | 0.59 |
| rs3097649 | 6 | 33056962 | HLA-DPB2 | 7.56E-05 | 1.82 | 2.84E-03 | 1.82 |
| rs3097650 | 6 | 33057440 | HLA-DPB2 | 9.65E-05 | 0.53 | 3.06E-02 | 0.64 |
| rs3117225 | 6 | 33057711 | HLA-DPB2 | 3.55E-05 | 0.50 | 1.22E-02 | 0.59 |
| rs3097652 | 6 | 33057835 | HLA-DPB2 | 4.99E-05 | 0.51 | 6.95E-03 | 0.56 |
| rs2068204 | 6 | 33058718 | HLA-DPB2 | 5.94E-05 | 1.83 | 3.27E-03 | 1.80 |
| rs10484569 | 6 | 33058952 | HLA-DPB2 | 9.50E-05 | 1.81 | 2.45E-03 | 1.84 |
| rs4282438 | 6 | 33072172 | HLA-DPB2 | 7.15E-05 | 1.82 | 2.84E-03 | 1.82 |
| rs2064476 | 6 | 33073322 | HLA-DPB2 | 7.53E-05 | 0.52 | 1.34E-02 | 0.59 |
| rs9368752 | 6 | 33073904 | HLA-DPB2 | 8.68E-05 | 1.81 | 7.70E-03 | 1.70 |
| rs9296080 | 6 | 33076539 | HLA-DPB2 | 8.16E-05 | 1.81 | 2.84E-03 | 1.82 |
| rs12174662 | 6 | 33079385 | HLA-DPB2 | 5.74E-05 | 1.84 | 6.68E-03 | 1.72 |
| rs9380344 | 6 | 33079689 | HLA-DPB2 | 9.86E-05 | 1.80 | 2.87E-03 | 1.83 |
| rs9348906 | 6 | 33080360 | HLA-DPB2 | 6.29E-05 | 1.82 | 2.48E-03 | 1.86 |
| rs7970095 | 12 | 31822256 | AMN1 | 5.15E-05 | 0.34 | 1.21E-02 | 0.44 |
| rs7494025 | 14 | 31720687 | HEATR5A | 1.35E-05 | 0.11 | 4.63E-02 | 0.36 |
| rs12595417 | 15 | 25793489 | LINC02250 | 1.82E-05 | 1.87 | 1.87E-03 | 0.52 |
| rs2063998 | 21 | 40816185 | SH3BGR | 2.01E-05 | 2.01 | 4.60E-02 | 1.52 |

Supplementary Table 2. Associations of HLA class II alleles with response to the Heptavax-II vaccine in a comparison between poor-responders (Group_0) and responders (Group_1 + Group_2)

| Haplotype/Allele |  | Group_0 | |  | Group_1 + Group_2 | |  | Chi P |  | OR | 95% CI | |
| --- | --- | --- | --- | --- | --- | --- | --- | --- | --- | --- | --- | --- |
|  |  | count | % |  | count | % |  |  |  |  | Lower | Upper |
| DRB1*01:01-DQB1*05:01 |  | 3 | 2.3 |  | 58 | 5.9 |  | 8.35E-02 |  | 0.37 | 0.11 | 1.19 |
| DRB1*04:05-DQB1*04:01 |  | 40 | 30.3 |  | 187 | 19.1 |  | 2.79E-03 |  | 1.84 | 1.23 | 2.75 |
| DRB1*08:03-DQB1*06:01 |  | 5 | 3.8 |  | 66 | 6.7 |  | 1.92E-01 |  | 0.54 | 0.22 | 1.38 |
| DRB1*09:01-DQB1*03:03 |  | 16 | 12.1 |  | 161 | 16.5 |  | 2.01E-01 |  | 0.70 | 0.40 | 1.21 |
| DRB1*13:02-DQB1*06:04 |  | 6 | 4.5 |  | 37 | 3.8 |  | 6.70E-01 |  | 1.21 | 0.50 | 2.93 |
| DRB1*15:01-DQB1*06:02 |  | 7 | 5.3 |  | 61 | 6.2 |  | 6.74E-01 |  | 0.84 | 0.38 | 1.88 |
| DRB1*15:02-DQB1*06:01 |  | 14 | 10.6 |  | 131 | 13.4 |  | 3.72E-01 |  | 0.77 | 0.43 | 1.38 |
| DPB1*02:01 |  | 20 | 15.9 |  | 213 | 23.6 |  | 5.33E-02 |  | 0.61 | 0.37 | 1.01 |
| DPB1*03:01 |  | 6 | 4.8 |  | 47 | 5.2 |  | 8.35E-01 |  | 0.91 | 0.38 | 2.18 |
| DPB1*04:02 |  | 6 | 4.8 |  | 80 | 8.8 |  | 1.20E-01 |  | 0.52 | 0.22 | 1.21 |
| **DPB1*05:01** |  | **76** | **60.3** |  | **389** | **43.0** |  | **2.59E-04** |  | **2.01** | **1.38** | **2.94** |
| DPB1*09:01 |  | 12 | 9.5 |  | 99 | 11.0 |  | 6.28E-01 |  | 0.86 | 0.46 | 1.61 |

The estimated DRB1-DQB1 haplotype frequencies over 5.0% and DPB1 allele frequencies over 5.0% in responders (Group_1 + Group_2) are shown. The result of DRB1*13:02-DQB1*06:04 was added for discussion in the paper. The total number of poor-responders and responders was 63 and 452, respectively, for the DPB1 allele and 66 and 489, respectively, for the DRB1-DQB1 haplotype. To set the family-wise error rate to be less than 5% in HLA association analysis for the haplotype or allele, the threshold of the P value was determined by the permutation test (P <0.00173 for the DRB1-DQB1 haplotype and P <0.00670 for the DPB1 allele). P values and odds ratios (OR) were calculated by Pearson’s chi-square test for presence vs. absence of each allele. P values and OR that were statistically significant after correction are indicated in bold.

Supplementary Table 3. Comparison of odds ratios between Heptavax-II and Bimmugen for 13 HLA class II alleles with P <0.05 in the association test of Bimmugen (Group_0 vs. Group_2)

|  | Allele | P value | OR Bimmugen | OR Heptavax-II |
| --- | --- | --- | --- | --- |
| *HLA-DRB1* | *01:01* | 4.05E-03 | 0.21 | 0.31 |
|  | *04:05* | 2.57E-09 | 2.85 | 2.55 |
|  | *08:02* | 2.07E-02 | 0.28 | 0.76 |
|  | *08:03* | 3.62E-04 | 0.19 | 0.45 |
|  | *14:06* | 4.82E-04 | 3.61 | 2.57 |
|  | *15:01* | 7.00E-04 | 0.17 | 0.64 |
| *HLA-DQB1* | *04:01* | 5.15E-09 | 2.79 | 2.55 |
|  | *05:01* | 2.73E-03 | 0.20 | 0.28 |
|  | *06:01* | 2.36E-02 | 0.60 | 0.65 |
|  | *06:02* | 8.92E-04 | 0.18 | 0.72 |
| *HLA-DPB1* | *04:01* | 1.98E-02 | 0.22 | 0.62 |
|  | *04:02* | 3.26E-05 | 0.13 | 0.46 |
|  | *05:01* | 5.09E-07 | 2.18 | 2.03 |

Supplementary Table 4. Associations of *HLA* class II genes in individuals with response (HBsAb >10 mIU/mL) to Heptavax-II and Bimmugen vaccines.

| Haplotype/Allele |  | Heptavax-II | |  | Bimmugen | |  | Chi P |  | OR | 95% CI | |
| --- | --- | --- | --- | --- | --- | --- | --- | --- | --- | --- | --- | --- |
|  |  | count | % |  | count | % |  |  |  |  | Lower | Upper |
| *DRB1*01:01-DQB1*05:01* |  | 58 | 5.9 |  | 121 | 6.0 |  | 9.43E-01 |  | 0.99 | 0.72 | 1.36 |
| ***DRB1*04:05-DQB1*04:01*** |  | **187** | **19.1** |  | **278** | **13.8** |  | **1.52E-04** |  | **1.48** | **1.21** | **1.81** |
| *DRB1*08:03-DQB1*06:01* |  | 66 | 6.7 |  | 175 | 8.7 |  | 6.95E-02 |  | 0.76 | 0.57 | 1.02 |
| *DRB1*09:01-DQB1*03:03* |  | 161 | 16.5 |  | 283 | 14.0 |  | 7.82E-02 |  | 1.21 | 0.98 | 1.49 |
| *DRB1*13:02-DQB1*06:04* |  | 37 | 3.8 |  | 122 | 6.0 |  | 9.59E-03 |  | 0.61 | 0.42 | 0.89 |
| *DRB1*15:01-DQB1*06:02* |  | 61 | 6.2 |  | 155 | 7.7 |  | 1.52E-01 |  | 0.80 | 0.59 | 1.09 |
| *DRB1*15:02-DQB1*06:01* |  | 131 | 13.4 |  | 222 | 11.0 |  | 5.67E-02 |  | 1.25 | 0.99 | 1.58 |
| *DPB1*02:01* |  | 213 | 23.6 |  | 469 | 23.2 |  | 8.50E-01 |  | 1.02 | 0.85 | 1.23 |
| *DPB1*03:01* |  | 47 | 5.2 |  | 107 | 5.3 |  | 9.08E-01 |  | 0.98 | 0.69 | 1.39 |
| *DPB1*04:02* |  | 80 | 8.8 |  | 195 | 9.7 |  | 4.86E-01 |  | 0.91 | 0.69 | 1.19 |
| *DPB1*05:01* |  | 389 | 43.0 |  | 833 | 41.3 |  | 3.75E-01 |  | 1.07 | 0.92 | 1.26 |
| *DPB1*09:01* |  | 99 | 11.0 |  | 204 | 10.1 |  | 4.90E-01 |  | 1.09 | 0.85 | 1.41 |

The estimated DRB1-DQB1 haplotype frequencies over 5.0% and DPB1 allele frequencies over 5.0% in individuals vaccinated with Bimmugen are shown. The total number of responders for Heptavax-II and Bimmugen was 452 and 1,009, respectively, for the DPB1 allele and 489 and 1,009, respectively, for the DRB1-DQB1 haplotype. To set the family-wise error rate to be less than 5% in HLA association analysis for the haplotype or allele, the threshold of P value was determined by the permutation test (P <0.00329 for the DRB1-DQB1 haplotype and P <0.00662 for the DPB1 allele). P values and odds ratios (OR) were calculated by Pearson’s chi-square test for presence vs. absence of each allele. P values and OR that were statistically significant after correction are indicated in bold.

Supplementary Table 5. Associations of *HLA* class II genes in individuals with poor response (HBsAb ≤10 mIU/mL) to Heptavax-II and Bimmugen vaccines.

| Haplotype/Allele |  | Heptavax-II | |  | Bimmugen | |  | Chi P |  | OR | 95% CI | |
| --- | --- | --- | --- | --- | --- | --- | --- | --- | --- | --- | --- | --- |
|  |  | count | % |  | count | % |  |  |  |  | Lower | Upper |
| *DRB1*04:05-DQB1*04:01* |  | 40 | 30.3 |  | 53 | 28.2 |  | 6.82E-01 |  | 1.11 | 0.68 | 1.81 |
| *DRB1*09:01-DQB1*03:03* |  | 16 | 12.1 |  | 34 | 18.1 |  | 1.48E-01 |  | 0.62 | 0.33 | 1.19 |
| *DRB1*14:06-DQB1*03:01* |  | 4 | 3.0 |  | 10 | 5.3 |  | 3.24E-01 |  | 0.56 | 0.17 | 1.81 |
| *DRB1*15:02-DQB1*06:01* |  | 14 | 10.6 |  | 21 | 11.2 |  | 8.74E-01 |  | 0.94 | 0.46 | 1.93 |
| *DPB1*02:01* |  | 20 | 15.9 |  | 43 | 22.9 |  | 1.29E-01 |  | 0.64 | 0.35 | 1.14 |
| *DPB1*05:01* |  | 76 | 60.3 |  | 108 | 57.4 |  | 6.13E-01 |  | 1.13 | 0.71 | 1.78 |
| *DPB1*09:01* |  | 12 | 9.5 |  | 20 | 10.6 |  | 7.49E-01 |  | 0.88 | 0.42 | 1.88 |

The estimated DRB1-DQB1 haplotype frequencies over 5.0% and DPB1 allele frequencies over 5.0% in individuals vaccinated with Bimmugen are shown. The total number of poor-responders for Heptavax-II and Bimmugen was 63 and 94, respectively, for the DPB1 allele and 66 and 94, respectively, for the DRB1-DQB1 haplotype. To set the family-wise error rate to be less than 5% in HLA association analysis for the haplotype or allele, the threshold of P value was determined by the permutation test (P <0.0113 for the DRB1-DQB1 haplotype and P <0.00274 for the DPB1 allele). P values and odds ratios (OR) were calculated by Pearson’s chi-square test for presence vs. absence of each allele. P values and OR that were statistically significant after correction are indicated in bold.

Supplementary Table 6. *HLA-DRB1-DQB1* haplotype frequency comparison between healthy individuals and high-responders for Heptavax-II and Bimmugen inoculation

| Haplotype |  | Healthy individuals  (2n=4,562) | |  | vs. Heptavax-II (2n=610) | | | | | |  | vs. Bimmugen (2n=1,372) | | | | | |
| --- | --- | --- | --- | --- | --- | --- | --- | --- | --- | --- | --- | --- | --- | --- | --- | --- | --- |
|  |  |  |  |  | count | % | Chi P | OR | 95% CI | |  | count | % | Chi P | OR | 95% CI | |
|  |  | count | % |  |  |  |  |  | Lower | Upper |  |  |  |  |  | Lower | Upper |
| *DRB1*01:01-DQB1*05:01* |  | 290 | 6.4 |  | 41 | 6.7 | 7.30E-01 | 1.06 | 0.76 | 1.49 |  | 97 | 7.1 | 3.48E-01 | 1.12 | 0.88 | 1.42 |
| *DRB1*04:05-DQB1*04:01* |  | 609 | 13.3 |  | 105 | 17.2 | 9.37E-03 | 1.35 | 1.08 | 1.69 |  | 166 | 12.1 | 2.28E-01 | 0.89 | 0.74 | 1.07 |
| *DRB1*08:03-DQB1*06:01* |  | 377 | 8.3 |  | 47 | 7.7 | 6.36E-01 | 0.93 | 0.68 | 1.27 |  | 139 | 10.1 | 3.14E-02 | 1.25 | 1.02 | 1.54 |
| *DRB1*09:01-DQB1*03:03* |  | 686 | 15.0 |  | 98 | 16.1 | 5.06E-01 | 1.08 | 0.86 | 1.36 |  | 183 | 13.3 | 1.19E-01 | 0.87 | 0.73 | 1.04 |
| *DRB1*13:02-DQB1*06:04* |  | 291 | 6.4 |  | 17 | 2.8 | **4.30E-04** | **0.42** | **0.26** | **0.69** |  | 93 | 6.8 | 5.98E-01 | 1.07 | 0.84 | 1.36 |
| *DRB1*15:01-DQB1*06:02* |  | 336 | 7.4 |  | 42 | 6.9 | 6.69E-01 | 0.93 | 0.67 | 1.30 |  | 116 | 8.5 | 1.82E-01 | 1.16 | 0.93 | 1.45 |
| *DRB1*15:02-DQB1*06:01* |  | 507 | 11.1 |  | 78 | 12.8 | 2.20E-01 | 1.17 | 0.91 | 1.51 |  | 139 | 10.1 | 3.06E-01 | 0.90 | 0.74 | 1.10 |

The estimated DRB1-DQB1 haplotype frequencies over 5.0% in healthy individuals are shown. To set the family-wise error rate to be less than 5% in HLA haplotype association analysis for Heptavax-II or Bimmugen, the threshold of P value was determined by the permutation test (P <0.00166 for Heptavax-II and P <0.00225 for Bimmugen). P values and odds ratios (OR) were calculated by Pearson’s chi-square test for presence vs. absence of each allele. P values and OR that were statistically significant after correction are indicated in bold.

Supplementary Table 7. 226 amino acids (aa) sequences of the HB vaccines

|  | aa sequence (N => C) |
| --- | --- |
| Heptavax-II | MENITSGFLGPLLVLQAGFFLLTRILTIPQSLDSWWTSLNFLGGSPVCLGQNSQSPTSNHSPTSCPPICPGYRWMCLRRFIIFLFILLLCLIFLLVLLDYQGMLPVCPLIPGSTTTSTGPCKTCTTPAQGNSM**FPSCCCTKPTDGNCT**CIPIPSSWAFAKYLWEWASVRFSWLSLLVPFVQWFVGLSPTVWLSAIWMMWYWGPSLYSIVSPFIPLLPIFFCLWVYI |
| Bimmugen | MENTTSGFLGPLLVLQAGFFLLTRILTIPQSLDSWWTSLNFLGGAPTCPGQNSQSPTSNHSPTSCPPICPGYRWMCLRRFIIFLFILLLCLIFLLVLLDYQGMLPVCPLLPGTSTTSTGPCKTCTIPAQGTSM**FPSCCCTKPSDGNCT**CIPIPSSWAFARFLWEWASVRFSWLSLLVPFVQWFVGLSPTVWLSVIWMMWYWGPSLYNILSPFLPLLPIFFCLWVYI |

Peptide consisting of 15 amino acids at positions 134 to 148 are indicated in bold.

Supplementary Table 8. Recognition of vaccine-derived peptides by HLA-DRB1*04:05

| Vaccine | start | end | peptide | method | core | IC50 | rank |
| --- | --- | --- | --- | --- | --- | --- | --- |
| Heptavax-II | 208 | 222 | IVSPFIPLLPIFFCL | NetMHCII 1.1 (SMM_align) | FIPLLPIFF | 50 | 0.62 |
|  | 206 | 220 | YSIVSPFIPLLPIFF | NetMHCII 1.1 (SMM_align) | IVSPFIPLL | 51 | 0.63 |
|  | 209 | 223 | VSPFIPLLPIFFCLW | NetMHCII 1.1 (SMM_align) | FIPLLPIFF | 52 | 0.65 |
|  | 207 | 221 | SIVSPFIPLLPIFFC | NetMHCII 1.1 (SMM_align) | FIPLLPIFF | 53 | 0.67 |
|  | 210 | 224 | SPFIPLLPIFFCLWV | NetMHCII 1.1 (SMM_align) | FIPLLPIFF | 53 | 0.67 |
|  | 202 | 216 | GPSLYSIVSPFIPLL | NetMHCII 1.1 (SMM_align) | LYSIVSPFI | 75 | 0.95 |
|  | 203 | 217 | PSLYSIVSPFIPLLP | NetMHCII 1.1 (SMM_align) | LYSIVSPFI | 77 | 0.97 |
|  | 201 | 215 | WGPSLYSIVSPFIPL | NetMHCII 1.1 (SMM_align) | LYSIVSPFI | 79 | 1 |
|  | 199 | 213 | WYWGPSLYSIVSPFI | NetMHCII 1.1 (SMM_align) | SLYSIVSPF | 81 | 1.04 |
|  | 200 | 214 | YWGPSLYSIVSPFIP | NetMHCII 1.1 (SMM_align) | LYSIVSPFI | 84 | 1.08 |
| Bimmugen | 203 | 217 | PSLYNILSPFLPLLP | NetMHCII 1.1 (SMM_align) | LYNILSPFL | 76 | 0.96 |
|  | 206 | 220 | YNILSPFLPLLPIFF | NetMHCII 1.1 (SMM_align) | LSPFLPLLP | 77 | 0.97 |
|  | 207 | 221 | NILSPFLPLLPIFFC | NetMHCII 1.1 (SMM_align) | FLPLLPIFF | 78 | 0.99 |
|  | 208 | 222 | ILSPFLPLLPIFFCL | NetMHCII 1.1 (SMM_align) | FLPLLPIFF | 81 | 1.04 |
|  | 209 | 223 | LSPFLPLLPIFFCLW | NetMHCII 1.1 (SMM_align) | FLPLLPIFF | 83 | 1.07 |
|  | 202 | 216 | GPSLYNILSPFLPLL | NetMHCII 1.1 (SMM_align) | LYNILSPFL | 84 | 1.08 |
|  | 210 | 224 | SPFLPLLPIFFCLWV | NetMHCII 1.1 (SMM_align) | FLPLLPIFF | 87 | 1.11 |
|  | 199 | 213 | WYWGPSLYNILSPFL | NetMHCII 1.1 (SMM_align) | GPSLYNILS | 89 | 1.14 |
|  | 201 | 215 | WGPSLYNILSPFLPL | NetMHCII 1.1 (SMM_align) | LYNILSPFL | 89 | 1.14 |
|  | 200 | 214 | YWGPSLYNILSPFLP | NetMHCII 1.1 (SMM_align) | LYNILSPFL | 92 | 1.18 |

Supplementary Table 9. Recognition of vaccine-derived peptides by HLA-DRB1*13:02

| Vaccine | start | end | peptide | method | core | IC50 | rank |
| --- | --- | --- | --- | --- | --- | --- | --- |
| Heptavax-II | 35 | 49 | WWTSLNFLGGSPVCL | NetMHCII 1.1 (SMM_align) | LNFLGGSPV | 576 | 14.36 |
|  | 37 | 51 | TSLNFLGGSPVCLGQ | NetMHCII 1.1 (SMM_align) | LNFLGGSPV | 582 | 14.47 |
|  | 36 | 50 | WTSLNFLGGSPVCLG | NetMHCII 1.1 (SMM_align) | LNFLGGSPV | 589 | 14.61 |
|  | 202 | 216 | GPSLYSIVSPFIPLL | NetMHCII 1.1 (SMM_align) | LYSIVSPFI | 654 | 15.83 |
|  | 203 | 217 | PSLYSIVSPFIPLLP | NetMHCII 1.1 (SMM_align) | LYSIVSPFI | 678 | 16.28 |
|  | 201 | 215 | WGPSLYSIVSPFIPL | NetMHCII 1.1 (SMM_align) | LYSIVSPFI | 687 | 16.44 |
|  | 199 | 213 | WYWGPSLYSIVSPFI | NetMHCII 1.1 (SMM_align) | SLYSIVSPF | 696 | 16.6 |
|  | 200 | 214 | YWGPSLYSIVSPFIP | NetMHCII 1.1 (SMM_align) | LYSIVSPFI | 719 | 17.02 |
|  | 38 | 52 | SLNFLGGSPVCLGQN | NetMHCII 1.1 (SMM_align) | FLGGSPVCL | 874 | 19.51 |
|  | 39 | 53 | LNFLGGSPVCLGQNS | NetMHCII 1.1 (SMM_align) | FLGGSPVCL | 886 | 19.69 |
| Bimmugen | 202 | 216 | GPSLYNILSPFLPLL | NetMHCII 1.1 (SMM_align) | LYNILSPFL | 748 | 17.5 |
|  | 201 | 215 | WGPSLYNILSPFLPL | NetMHCII 1.1 (SMM_align) | LYNILSPFL | 793 | 18.24 |
|  | 203 | 217 | PSLYNILSPFLPLLP | NetMHCII 1.1 (SMM_align) | LYNILSPFL | 796 | 18.29 |
|  | 199 | 213 | WYWGPSLYNILSPFL | NetMHCII 1.1 (SMM_align) | SLYNILSPF | 871 | 19.47 |
|  | 180 | 194 | VQWFVGLSPTVWLSV | NetMHCII 1.1 (SMM_align) | WFVGLSPTV | 896 | 19.85 |
|  | 200 | 214 | YWGPSLYNILSPFLP | NetMHCII 1.1 (SMM_align) | LYNILSPFL | 911 | 20.06 |
|  | 181 | 195 | QWFVGLSPTVWLSVI | NetMHCII 1.1 (SMM_align) | LSPTVWLSV | 1214 | 24.13 |
|  | 182 | 196 | WFVGLSPTVWLSVIW | NetMHCII 1.1 (SMM_align) | LSPTVWLSV | 1262 | 24.71 |
|  | 184 | 198 | VGLSPTVWLSVIWMM | NetMHCII 1.1 (SMM_align) | LSPTVWLSV | 1495 | 27.36 |
|  | 204 | 218 | SLYNILSPFLPLLPI | NetMHCII 1.1 (SMM_align) | LYNILSPFL | 1658 | 28.99 |

Supplementary Table 10. Clinical information of 555 individuals who were vaccinated with a recombinant absorbed HB vaccine (Heptavax^R^-II, MSD K.K., Tokyo, Japan)

|  | Group_0 | Group_1 | Group_2 |
| --- | --- | --- | --- |
| HbsAb (mIU/mL) | HBsAb ≤10 | 10 < HBsAb <100 | HBsAb ≥100 |
| total number | 66 | 184 | 305 |
| male/female | 28/38 | 74/110 | 85/220 |
| age (mean±SD) | 32.0±12.9 | 34.4±11.6 | 30.0±9.4 |
| HBsAb (mean±SD) | 2.8±3.1 | 45.7±26.1 | 948±2527 |
| HBcAb (mean±SD) | 0.09±0.04 | 0.10±0.09 | 0.10±0.06 |
| #vaccination |  |  |  |
| naive | 18 | 63 | 85 |
| 1-7 | 41 | 94 | 168 |
| n/a | 7 | 27 | 52 |

Supplementary Table 11. Clinical information of 1,193 individuals who were vaccinated with a recombinant absorbed HB vaccine (Bimmugen^R^, Kaketsuken, Kumamoto, Japan). This table is a modified version of the table from our previous study (reference 9).

|  | Group_0 | Group_1 | Group_2 |
| --- | --- | --- | --- |
| HbsAb (mIU/mL) | HBsAb ≤10 | 10 < HBsAb <100 | HBsAb ≥100 |
| total number | 107 | 351 | 735 |
| male/female | 48/59 | 163/188 | 230/505 |
| age (mean±SD) | 34.4±12.7 | 30.2±10.9 | 26.3±9.0 |
| HBsAb (mean±SD) | 4.3±3.3 | 49.4±26.4 | 1872±6782 |
| HBcAb (mean±SD) | 0.09±0.05 | 0.08±0.06 | 0.09±0.07 |
| #vaccination |  |  |  |
| naive | 36 | 172 | 403 |
| 1-10 | 41 | 93 | 204 |
| n/a | 30 | 83 | 128 |
